# Neuroimmunomodulation of vagus nerve stimulation and the therapeutic implications

**DOI:** 10.3389/fnagi.2023.1173987

**Published:** 2023-07-06

**Authors:** Yi-Ting Fang, Ye-Ting Lin, Wu-Lung Tseng, Philip Tseng, Gia-Linh Hua, Ying-Jui Chao, Yi-Jen Wu

**Affiliations:** ^1^Department of Neurology, National Cheng Kung University Hospital, College of Medicine, National Cheng Kung University, Tainan, Taiwan; ^2^Department of Surgery, National Cheng Kung University Hospital, College of Medicine, National Cheng Kung University, Tainan, Taiwan; ^3^Institute of Clinical Medicine, College of Medicine, National Cheng Kung University, Tainan, Taiwan; ^4^Cross College Elite Program, National Cheng Kung University, Tainan, Taiwan; ^5^Research Center for Mind, Brain and Learning, National Chengchi University, Taipei, Taiwan; ^6^School of Medicine, College of Medicine, National Cheng Kung University, Tainan, Taiwan

**Keywords:** vagus nerve, vagus nerve stimulation, non-invasive VNS, neuroimmunomodulation, cholinergic anti-inflammatory pathway, inflammatory disease

## Abstract

Vagus nerve stimulation (VNS) is a technology that provides electrical stimulation to the cervical vagus nerve and can be applied in the treatment of a wide variety of neuropsychiatric and systemic diseases. VNS exerts its effect by stimulating vagal afferent and efferent fibers, which project upward to the brainstem nuclei and the relayed circuits and downward to the internal organs to influence the autonomic, neuroendocrine, and neuroimmunology systems. The neuroimmunomodulation effect of VNS is mediated through the cholinergic anti-inflammatory pathway that regulates immune cells and decreases pro-inflammatory cytokines. Traditional and non-invasive VNS have Food and Drug Administration (FDA)-approved indications for patients with drug-refractory epilepsy, treatment-refractory major depressive disorders, and headaches. The number of clinical trials and translational studies that explore the therapeutic potentials and mechanisms of VNS is increasing. In this review, we first introduced the anatomical and physiological bases of the vagus nerve and the immunomodulating functions of VNS. We covered studies that investigated the mechanisms of VNS and its therapeutic implications for a spectrum of brain disorders and systemic diseases in the context of neuroimmunomodulation.

## 1. Introduction

Vagus nerve stimulation (VNS) is a modern technique that delivers electrical stimulation to the vagus nerve. Traditional VNS is the earliest model of VNS in which the stimulation electrode cuff is invasively implanted at the left cervical vagus nerve, and the electrical generator is embedded in the subcutaneous space of the left anterior chest (Table 1). Traditional VNS is Food and Drug Administration (FDA) approved as an adjunctive therapy for patients with drug-resistant epilepsy (DRE) and treatment-resistant depression (Ben-Menachem, 2002; Young et al., 2020). Non-invasive VNS (nVNS) has been developed to extracorporeally deliver electrical stimulation to the vagus nerve and includes transcutaneous or percutaneous delivery to the cervical or auricular vagus nerve branches, which has been applied in human and animal studies for the treatment of a variety of diseases (Butt et al., 2020; Hilz, 2022) (Table 1). The FDA has approved transcutaneous cervical VNS (tcVNS) for refractory migraine and cluster headache (CH) (Silberstein et al., 2016a). Currently, VNS is an emerging technique being investigated in clinical trials and experimental studies to explore its underlying mechanisms and therapeutic effects for a broad spectrum of general medical disorders beyond neuropsychiatric disorders (Downes et al., 2023). The mechanism of action of VNS is not fully understood (Henry, 2002; Wang Y. et al., 2021). It is believed to be mediated through the activation of vagal afferents and efferents projecting upward to the brain and downward to internal organs. The vagal afferents signal the activation of brainstem nuclei and the relayed cortical projections, leading to multiple effects on brain activities, neurotransmitters, and hypothalamic–pituitary–adrenal (HPA) axis-related endocrine functions. The vagal efferent fibers are widely distributed in internal organs, and transmit the communication of the nervous system with the immune system to modulate the neuroendocrine and neuroimmunology, mainly through the cholinergic anti-inflammatory pathway (CAP) (Manta et al., 2012; Thrivikraman et al., 2013; Tarn et al., 2019). CAP-mediated neuroimmunomodulation underlies the therapeutic effects of VNS. The modulations of neural circuit, neuroendocrine, and neuroimmune responses are thought to be underpinning the VNS therapeutic effects (Kelly et al., 2022). In this review, we introduced the anatomical and physiological bases of vagus nerve associated with immune systems, the immunomodulation by VNS, and its therapeutic implications.

**Table 1:** VNS modality types.

**Invasiveness**	**Modality**	**Animal/human**	**Disease/function**	**Reference**
Invasive	VNS: Implanted electrode coil at the left cervical vagus nerve (traditional VNS)	Human—FDA approved	Drug-resistant epilepsy	Morris and Mueller (1999) Labar et al. (1999) Morris et al. (2013) Kawai et al. (2017)
			Treatment-resistant depression	Rush et al. (2005a) George et al. (2005) Rush et al. (2005b)
			Post-ischemic stroke rehabilitation	Dawson et al. (2021)
		Human—Clinical trial	Verbal memory in DRE	Mertens et al. (2022)
			Electrocardiograms in DRE with VNS	Verrier et al. (2016)
			Ictal tachycardia-triggered VNS for DRE	Fisher et al. (2016)
			Tinnitus	De Ridder et al. (2015) Tyler et al. (2017)
			Fibromyalgia	Lange et al. (2011)
			Generalized anxiety disorder	George et al. (2008)
			Rheumatoid arthritis	Genovese et al. (2020)
		Animal	Anti-inflammation in the brain	Mihaylova et al. (2012) Meneses et al. (2016) Namgung et al. (2022) Caravaca et al. (2022)
			Parkinson's disease	Farrand et al. (2017) Farrand et al. (2020)
			Gulf War illness	Nizamutdinov et al. (2018) Venkatasamy et al. (2021) Iannucci et al. (2022)
			Colitis and cerebral cortical microinfarct	Chen et al. (2018)
			Chemotherapy-induced peripheral neuropathy	Zhang et al. (2020)
			Cerebral ischemia and pyroptosis	Tang et al. (2022)
			Spinal cord injury	Chen et al. (2022)
	Transvenous VNS: Stimulation catheter inserted in the left internal jugular vein at spinal level C5–C7	Human – Clinical trial	Anti-inflammation	Kox et al. (2015)
Non-invasive VNS (nVNS)	Transcutaneous cervical VNS (tcVNS): Stimulation applied via the neck skin	Human—FDA approved	Acute treatment of episodic cluster headache	Silberstein et al. (2016b) Goadsby et al. (2018)
			Acute treatment of episodic migraine	Tassorelli et al. (2018) Grazzi et al. (2017)
			Prevention of chronic cluster headache	Gaul et al. (2016)
			Prevention of episodic or chronic migraine	Silberstein et al. (2016a) Diener et al. (2019) Najib et al. (2022)
		Human—Clinical trial	Acute stroke	Arsava et al. (2022)
			Atrial fibrillation	Stavrakis et al. (2020)
			Post-traumatic stress disorder	Wittbrodt et al. (2021)
			Opioid use disorder	Gazi et al. (2022)
			COVID-19-related inflammation	Tornero et al. (2022)
			Painful chronic pancreatitis	Muthulingam et al. (2021)
		Animal	Migraine	Liu et al. (2022)
	Percutaneous cervical VNS: Needle electrode insertion at the carotid sheath	Animal	Anti-inflammation and inflammation-related cognitive dysfunction	Huffman et al. (2019)
	Transcutaneous auricular VNS (taVNS): Stimulation applied on the left ear concha	Human–Clinical trial	Improved verbal memory in patients with DRE	Mertens et al. (2022)
			Tinnitus	Kreuzer et al. (2014)
			Post-stroke rehabilitation	Redgrave et al. (2018) Chang et al. (2021)
			Improved poor feeding of infant	Badran et al. (2020)
			Type 2 diabetes mellitus Glucotropic and orexigenic hormones	Gomolka et al. (2018) Kozorosky et al. (2022)
			Fibromyalgia	Paccione et al. (2022)
			Major depressive disorder	Garcia et al. (2021)
			Opioid use disorder	Tirado et al. (2022)
			Rheumatoid arthritis	Addorisio et al. (2019)
			Post-operative inflammatory response	Salama et al. (2020)
			Erosive hand osteoarthritis	Courties et al. (2022)
			Idiopathic nephrotic syndrome	Merchant et al. (2022)
			Constipation-predominant irritable bowel syndrome	Shi et al. (2021)
			Prader–Willi syndrome	Manning et al. (2019)
			Heart rate augmentation	Badran et al. (2018)
			Motor performance	Hatik et al. (2022)
			Sleep	Jackowska et al. (2022)
			Gastric motility	Steidel et al. (2021)
			Intestinal barrier dysfunction	Mogilevski et al. (2022)
	Self-adsorption conductive magnet in the bilateral cavity of auricular concha	Animal	Antidepression and neuroinflammation	Guo et al. (2020)
	Percutaneous auricular VNS: Acupuncture needles inserted at the left cavum concha	Animal	Anti-neuroinflammation and dopaminergic neurodegeneration of the substantia nigra	Jiang et al. (2018)
			Surgery-induced cognitive dysfunction	Cai et al. (2019)
			Hippocampal neuroinflammation and depression	Wang J. Y. et al. (2021)

## 2. Anatomical bases of the vagus nerve

The vagus nerve is the longest cranial nerve and is the 10th cranial nerve. It functions in bidirectional communication between the brain and internal organs and is involved in regulating the homeostasis of autonomic systems. The vagus nerve primarily comprises unmyelinated sensory afferent fibers, accounting for 80–90% of the nerve fiber, with the remaining 10–20% being myelinated efferent fibers (Foley and Dubois, 1937; Asala and Bower, 1986; Dolphin et al., 2022; Prescott and Liberles, 2022). Most known actions of the vagus nerve depend on the vagal efferent innervations that are distributed from the neck to the heart, lung, spleen, and gastrointestinal (GI) organs (Wang and Powley, 2007). The vagus efferent fibers originate from the nucleus ambiguous (NAm) and dorsal motor nucleus of the vagus nerve (DMV), both located in the medulla. Vagal somatic motor fibers arise from the NAm and terminate at the laryngeal and pharyngeal muscles that control the soft palate and function in speech and swallowing. Meanwhile, the NAm contributes to preganglionic parasympathetic fibers of the vagal cardiac branches that innervate the postganglionic parasympathetic neurons to decrease the heart rate (Machado and Brody, 1988). The DMV gives rise to the preganglionic parasympathetic fibers that are distributed throughout the lung and GI tract. These fibers primarily innervate the postganglionic neurons located in the myenteric plexuses of the muscularis propria and, to a lesser extent, the submucosal plexus and function in muscular contraction and glandular secretion (Berthoud et al., 1990). The majority of the vagus nerve fibers are sensory afferent fibers that transmit sensory information from internal organs to the central nervous system. The vagal afferent signals arise from the tension receptors of the stomach and esophagus, chemoreceptors and mechanoreceptors from the GI mucosa (Cirillo et al., 2022). And sensory inputs from the liver and pancreas project to the nucleus tractus solitarii (NTS) via the vagal nodose ganglion, with secondary projections to the raphe nucleus, locus coeruleus (LC), thalamus, hypothalamus, amygdala, and cerebral cortex (Nemeroff et al., 2006; Decarie-Spain et al., 2023). The LC is a crucial structure for norepinephrine release (Berger et al., 2021) and the raphe nucleus for widespread serotonergic innervations (Hornung, 2003). Therefore, the projections of the vagus nerve to the LC and raphe nucleus regulate the neurotransmitters norepinephrine and serotonin, respectively. The ascending bilateral vago-solitario-parabrachial pathways involve the central autonomic, reticular activating, and limbic systems. The ascending unilateral vago-trigemino-thalamocortical pathways project to the somatosensory system (Henry, 2002). Therefore, the vagal afferent input can activate several brainstem nuclei and circuits to affect multiple neuropsychological functions. The vagal afferent and efferent fibers function as the parasympathetic autonomic nervous system and bridge the communication between the central, cardiopulmonary, and enteric nervous systems. The vagus nerve allows bidirectional modulations between the brain and autonomic systems to influence cognition, emotion, enteroendocrine, and immunomodulation functions (Carabotti et al., 2015).

## 3. Neuroimmunomodulation of the vagus nerve and VNS

**Figure 1:**
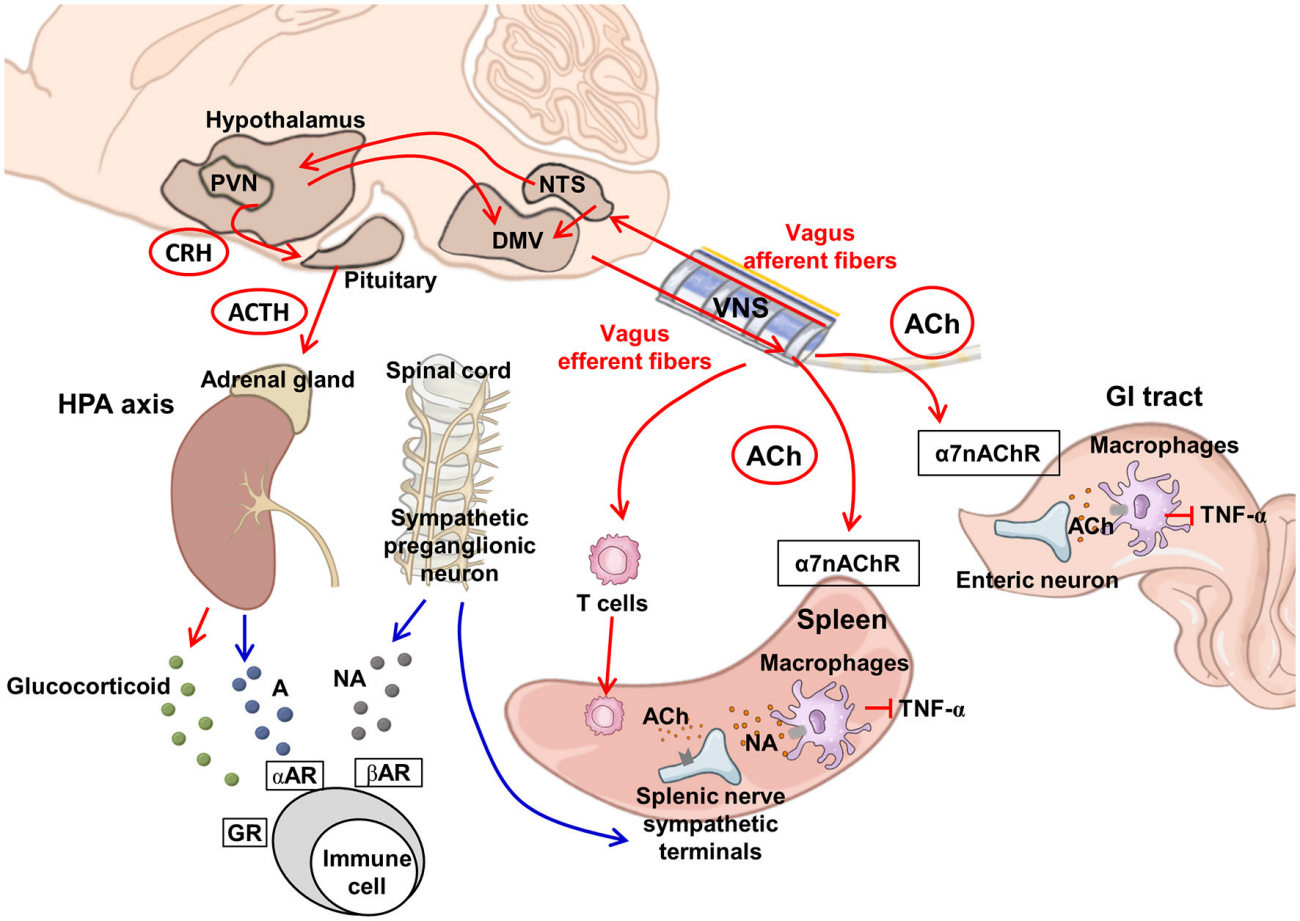
Proposed neuroimmunomodulation by vagus nerve stimulation. VNS can activate vagal afferent fibers projecting to the NTS, which relays to the PVN in the hypothalamus and DMV. The DMV transmits downward signals via vagal efferent fibers. The PVN activates the HPA axis, releasing glucocorticoids from the adrenal glands to exert an anti-inflammatory effect (Berthoud and Neuhuber, 2019). The sympathetic preganglionic neurons project to the splenic nerve. The splenic nerve sympathetic terminals can be modulated by ACh via the vagus-to-spleen circuit. In addition, the activation of ACh-synthesizing T cells can stimulate the sympathetic terminal of the splenic nerve to release NA and suppress macrophages to produce TNF-α (Martelli et al., 2014). Vagal efferent fibers in the gut innervate the postganglionic enteric neurons and suppress macrophage TNF-α production, via alpha-7 nicotinic receptors (Bonaz et al., 2017). The red arrow line indicates the vagus nerve pathway and regulation, whereas the blue line indicates the regulation by the sympathetic pathway. A, adrenaline; ACh, acetylcholine; ACTH, adrenocorticotrophic hormone; CRH, corticotrophin-releasing hormone; DMV, dorsal motor nucleus of the vagus; GR, glucocorticoid receptor; GI, gastrointestinal; HPA, hypothalamic–pituitary–adrenal; NA, noradrenaline; NTS, nucleus tractus solitarius; PVN, paraventricular nucleus at the hypothalamus; TNF-α, tumor necrosis factor-α; VNS, vagus nerve stimulation; α7nAChR, α-7-nicotinic ACh receptors; αAR, alpha-adrenergic receptor; βAR, beta-adrenergic receptor.

The vagal efferent route gives rise to the CAP, mediated through the release of acetylcholine (ACh) (Figure 1). ACh binding to the α-7-nicotinic ACh receptors (α7nAChRs) on macrophages inhibits tumor necrosis factor (TNF)-α synthesis and secretion and suppresses inflammation (Bonaz et al., 2016). The vagal efferent route synapses with the celiac ganglion, which relays the signal to T cells. As these T cells enter the spleen, they release ACh, which binds on the α7nAChR on the splenic sympathetic terminal nerves to release noradrenaline signaling to splenic macrophages to decrease the release of TNF-α (Martelli et al., 2014). Macrophages also respond to ACh when it binds to the α7nAChR, where ACh inhibits the macrophage TNF synthesis and reduces the levels of the inflammatory cytokine (Wang et al., 2003). The GI tract receives various antigens via food and liquid ingestion. The vagus nerve functions as the bridge for the brain–gut axis and has the primary role of immunomodulation in the GI tract. The vagal CAP synapses directly on the enteric neurons and modulates macrophages to reduce TNF-α synthesis (Berthoud and Neuhuber, 2019). The immunomodulation by the vagal afferent route is mediated through the HPA axis (Figure 1). Environmental stress and systemic pro-inflammatory cytokines can trigger the release of corticotropin-releasing factor (CRF) from the hypothalamus. CRF further stimulates the release of adrenocorticotropic hormone (ACTH) from the pituitary. After stimulation with ACTH, the adrenal medulla exerts the immunomodulating function by secreting cortisol, which acts on multiple systems, including the brain, bones, muscles, and body fat, as a response to stress (Mayer et al., 2014).

The vagus nerve has immunomodulatory functions through vagal efferent fibers mediated by the CAP and vagal afferent circuits mediated by the HPA axis. Therefore, stimulating the vagus nerve using VNS enhances vagus nerve-mediated anti-inflammatory effects and is applied in the treatment of multiple diseases with underlying inflammatory etiologies (Kelly et al., 2022). Typically, VNS exerts its immunomodulating effects via the CAP to reduce pro-inflammatory cytokines, activate microglia and macrophages, and alter the consequences of neuroinflammation (Figure 1). The activated vagus nerve by VNS increases the release of ACh, which binds to the α7nAChR in cytokine-producing immune cells and decreases the production of inflammatory cytokines. Wang et al. found that TNF synthesis was inhibited in wild-type mice treated with VNS; however, this inhibition was diminished in α7-deficient mice, suggesting that the anti-inflammatory action of VNS is mediated via ACh and the α7nAChR (Wang et al., 2003). In an ovine fetus with umbilical cord occlusion-induced ischemic injury, heart rate variability (HRV), which reflects vagus nerve activity, was positively correlated with plasma interleukin (IL)-1β levels and negatively correlated with white matter microglia cell counts. High-mobility group box protein 1 (HMGB1) is a DNA-binding protein, and its translocation functions to promote pro-inflammatory cytokines and induce microglial activation. The hypoxic insult increases HMGB1 translocation in α7nAChR^+^ microglia in a brain region-dependent manner. Higher HRV values are correlated with lower HMGB1 translocation and higher α7nAChR intensity in a brain region-specific manner, suggesting the CAP-mediated anti-inflammatory role of the vagus nerve in early perinatal hypoxic brain injury (Frasch et al., 2016). Stimulating the vagus nerve using VNS significantly reduces the levels of pro-inflammatory cytokines (IL-1β, IL-6, and TNF-α) and the percentage of CD11b^+^CD45^low^ microglia and CD11b^+^/CD45^high^ macrophages in the brain. In mice with lipopolysaccharide-induced inflammation treated with VNS, a significantly reduced level of ionized calcium-binding adapter molecule 1 (Iba-1) expression, implying a reduction in activated microglia, was observed following VNS in the brain under an inflammatory context (Meneses et al., 2016). In an endotoxemic rat model, Mihaylova et al. demonstrated that VNS decreased the plasma levels of interleukin (IL)-6, tumor necrosis factor (TNF)-α, interferon (IFN)-γ, IL-10, and cerebral hypoxia-inducible factor (HIF)-2α expression following sepsis and stabilized hemodynamic responses in the animals with sepsis (Mihaylova et al., 2012). Caravaca et al. reported the effect of VNS on lipid metabolomes during inflammation. In mice with iatrogenic peritonitis, the duration of inflammation resolution was significantly reduced, and efferocytosis was significantly increased in the mice treated with VNS. In supernatants of peritoneal exudates, IL-6 and IL-1β levels were significantly lower in VNS-treated mice. Mice treated with VNS had higher levels of specialized pro-resolving mediators (SPMs), particularly from the omega-3 docosahexaenoic (DHA) and n-3 docosapentaenoic acid (DPA) metabolomes, in the peritoneal exudates. VNS also shifted the ratio between pro-inflammatory and pro-resolving lipid mediators toward a pro-resolving profile. This effect was absent in mice deficient in 12/15-lipoxygenase (Alox15), a key enzyme in SPM biosynthesis. The significant VNS-mediated reduction of neutrophil numbers in peritoneal exudates was diminished in mice deficient in α7nAChR. These findings support the notion that the anti-inflammatory effect induced by VNS in peritonitis is likely mediated via α7nAChR and involves Alox15 (Caravaca et al., 2022). The HPA axis can be affected by VNS via vagal afferent pathways (Hosoi et al., 2000; Thrivikraman et al., 2013; Bonaz et al., 2021). A few *in vivo* evidence suggest the causal relationship between HPA axis regulation and VNS neuromodulation. The CRF-induced ACTH serum concentrations were decreased in normal rats treated with VNS (Chen et al., 2021). Similarly, a reduction of the over-elevated ACTH responses under CRF challenge tests was reported in patients with chronic depression following VNS (O'keane et al., 2005). Increased serum corticosterone levels, neuronal activation measured by c-Fos at NTS, and reduced food intake with body weight loss were observed in rats fed with high-fat diet following VNS treatment (De Herdt et al., 2009; Thrivikraman et al., 2013). The changes in CRF-induced ACTH responses and serum corticosterone support the notion that VNS modulates the neuroendocrine functions via the HPA axis.

## 4. Neuroinflammation modulation by VNS in animal models

VNS was applied in variable animal models of neuropsychiatric disorders to examine whether it can ameliorate the disorders through its immunomodulatory effects (Table 2).

**Table 2:** Neuroinflammation modulation by VNS in animal model.

**Authors**	**Year**	**Models**	**VNS setting**	**Physiology/function studied**
Mihaylova et al.	2012	Rat	2 mA, 2 Hz, pulse width 0.3 ms, 10 min, repeated every 45 min, total 4.5 h	Anti-inflammatory effect of VNS on cerebral microcirculatory integrity
Meneses et al.	2016	Rat	0.75 mA, 5 Hz, pulse width 2 ms, 30 s	Anti-inflammatory effects of VNS
Farrand et al.	2017	Rat	0.8 mA, 30 Hz, 100 μs, 500 ms, 15 biphasic pulses every 30 s, 30 min twice, 4 h apart, 10 days	Therapeutic potential of VNS in a rat PD model
Nizamutdinov et al.	2018	Rat	Amplitude n/a, 5 Hz, pulse width 1 ms, 30 s on, 4 min and 30 s off, for 2 or 4 weeks	Pain threshold restoration by VNS in a GWI rat model
Jiang et al.	2018	Rat	Percutaneous auricular VNS: 0.8 mA, 30 Hz, 500 ms train of 15 biphasic pulses every 30 s, 30 min, every 2 days for 8 days, acupuncture needles inserted into the left cavum concha	SN dopaminergic neurodegeneration, the associated neuroinflammation and immune responses in PD animals
Chen et al.	2018	Mice	0.5 mA, 5 Hz, 30 s every 5 min for 1 h	Effects on CMI by VNS in animals of CMI when exposed to colitis and the anti-inflammatory mechanisms
Huffman et al.	2019	Mice	Percutaneous cervical VNS: amplitude to reach 10% reduction in HR, 20 Hz, pulse width 300 ms, biphasic, charge-balanced pulses, 30 min, echo-guided needle electrode insertion at carotid sheath	Change of microglial activation and cognitive dysfunction following LPS endotoxinemia by VNS
Cai et al.	2019	Rat	Percutaneous auricular VNS: 0.5 mA, 20 Hz, 30 s train, 4 times, 5 min apart, acupuncture needles implanted on the left cavum concha	Changes of surgery-induced cognitive dysfunction in an aged rat model of POCD by VNS
Farrand et al.	2020	Rat	0.75 A, pulse width 250 μs, with (1) low VNS: 10 Hz, 30 min on, 23.5 h off (2) standard VNS: 20 Hz, 30 s on, 5 min off, 24 h/day (3) burst VNS: 300 Hz (10 pulses per burst), 19 s inter-burst interval, 24 h/day	Different VNS paradigms in a PD animal model
Guo et al.	2020	Rat	Transauricular VNS (taVNS): 2 mA, 15 Hz, 30 min, 28 days, self-adsorption conductive magnet fixed in bilateral cavity of the auricular concha	Anti-inflammatory effect of VNS in rats with depression and chronic somatic pain comorbidity
Zhang et al.	2020	Rat	1 V, 5 Hz, 2 ms, 30 s on, 5 min off, for 20 min	Changes of neuroinflammation and CIPN by VNS via CAP
Venkatasamy et al.	2021	Mice	Amplitude n.p., 5 Hz, pulse width 1 ms, 30 s on, 4 min 30 s off, continuous for 2 weeks	Chronic cognitive deficit and hippocampal astrocytosis in a rat model of GWI
Wang J. Y. et al.	2021	Rat	Percutaneous auricular VNS: EA apparatus at the auricular concha, 2 mA, 2 and 15 Hz, switched every s, 30 min/days, 21 days	Changes of hippocampal neuroinflammation and role of α7nAChR by taVNS
Liu et al.	2022	Rat	Transcutaneous cervical VNS on skin covering right vagal nerve, 3 parameters tested: (1) voltage: 1 V, 11.4 V, or 24.4 V, 25 Hz, 2 min (2) duration: 11.4 V, 25 Hz, 2 or 3 rounds of 2 min, 5 min apart, or single 6 min (3) chronic daily VNS: 11.4 V, 25 Hz, 2 rounds of 2 min, 5 min apart, 4 weeks vs. single VNS	Optimal nVNS paradigm for migraine
Namgung et al.	2022	Rat	Acute VNS: 10 mA, 5 Hz, pulse width 5 ms, 5 min Chronic VNS: 10 mA, 5 Hz, pulse width 5 ms, 5 min, 7 days	Regulation of pro-inflammatory cytokines in the hippocampus in a rat model of continuous stress
Tang et al.	2022	Rat	0.5 mA, 20 Hz, pulse width 0.5 ms, 30 s on and 5 min off, total 1h	Neuroprotective effects of VNS through α7nAChR-mediated inhibition of pyroptosis
Iannucci et al.	2022	Mice	Amplitude n/a, 5 Hz, 1 ms pulse width, 30 s on, 4 min 30 s off, total 2–4 weeks	Improvement of cognitive impairment and hippocampal dysfunction by VNS in a model of GWI
Chen et al.	2022	Rat	1.0 mA, 0.5 ms, 30 Hz, 10 min/day, 14 days	Efficacy and therapeutic mechanism of VNS in SCI
Caravaca et al.	2022	Mice	1 mA, 250 μs biphasic pulse, 50 s interphase delay, 10 Hz, 5 min	Resolution of inflammation by VNS

α7nAChR, α7 nicotinic acetylcholine receptor; CAP, cholinergic anti-inflammatory pathway; CIPN, chemotherapy-induced peripheral neuropathy; CMI, cerebral cortical microinfarct; EA, electroacupuncture; GWI, Gulf War Illness; LPS, lipopolysaccharide; n/a: not applicable; PD, Parkinson's disease; POCD, Post-operative cognition decline; SCI, spinal cord injury; SN, substantia nigra; VNS, vagus nerve stimulation.

### 4.1. Traditional VNS

#### 4.1.1. Parkinson's disease

Farrand et al. demonstrated that VNS significantly improved locomotion and increased the expression of tyrosine hydroxylase (TH) in the striatum, substantia nigra (SN), and LC in an animal model of Parkinson's disease induced by bilateral intrastriatal injection of dopaminergic neurotoxin 6-hydroxydopamine (6-OHDA). VNS decreased α-synuclein expression in the SN and glial markers in the SN and LC of lesioned rats. Furthermore, higher LC TH and lower SN Iba-1 were observed following VNS. Furthermore, they discovered that higher VNS frequencies, specifically microburst VNS, provided greater improvements in motor function, attenuation of TH-positive cell loss in the SN and LC, and norepinephrine concentration in the prefrontal cortex. Additionally, higher VNS frequencies resulted in lower intrastromal α-synuclein accumulation and glial density in the SN (Farrand et al., 2017, 2020).

#### 4.1.2. Cerebral ischemia

Tang et al. showed that VNS inhibited pyroptosis following a cerebral ischemic/reperfusion injury through the CAP, mediated by α7nAChR. The VNS-treated group showed decreased expressions of the nucleotide-binding domain, leucine-rich-containing family, pyrin domain-containing-3 (NLRP3), and its inflammasome, cleaved caspase-1, and N-terminal fragment of gasdermin D (GSDMD-N), which would cause cell membrane rupture and release of inflammatory cytokines. The apoptosis-associated speck-like protein containing a caspase-recruitment domain (ASC), the number of pyroptotic cells, and membrane pores were also decreased in VNS-treated animals. Furthermore, VNS downregulated IL-1β and IL-18 in brain tissues. Administration of an α7nAChR-agonist mimicked the VNS effects in terms of the improvement of neurological deficits, reduction of infarct volumes, and inhibition of neuronal pyroptosis after cerebral ischemia/reperfusion injury. The neuroprotection provided by VNS could be reversed by the administration of an α7nAChR antagonist (Tang et al., 2022).

#### 4.1.3. Colitis and inflammatory bowel disease

Cerebral microinfarction (CMI) was aggravated by colitis in mice with CMI treated with dextran sodium sulfate (DSS). In mice with CMI and colitis, VNS significantly reversed the increases in blood–brain barrier permeability, infarct volume, activation of Iba-1^+^ microglia and GFAP^+^ astrocytes, and TNF-α level in mice with colitis and CMI. Brain lesions in mice with colitis and CMI were significantly ameliorated when VNS was performed during acute colitis (Chen et al., 2018). VNS attenuated the systemic inflammatory response to endotoxins and intestinal inflammation in the animal model of inflammatory bowel disease (IBD). In animals with chronic colitis and IBD, VNS resulted in a reduction of inflammatory markers and an improvement in colitis symptoms (Sinniger et al., 2016; Breit et al., 2018; Bonaz et al., 2021).

#### 4.1.4. Depression and chronic stress

In rats with chronic stress, the levels of TNF-α, IL-1β, and IL-6 in the gastric and hippocampal tissues significantly decreased by VNS. After VNS treatment, Iba-1-labeled microglial cells in the hippocampus also showed changes in morphological features from activated inflammatory cells to normal shapes. VNS increased hippocampal expressions of α7nAChR, and pharmacological blockade of α7nAChR increased the production of TNF-α, IL-1β, and IL-6 and suppressed cholinergic anti-inflammatory activities mediated by VNS. Chronic VNS downregulated hippocampal expression of the active form of caspase 3 and 5-HT1A receptors and decreased the levels of TNF-α, IL-1β, and IL-6 in the gastric and hippocampal tissues. Furthermore, pain sensitivity and depressive-like behaviors were improved by chronic VNS (Namgung et al., 2022).

#### 4.1.5. Gulf War illness

The Gulf War illness comprises multiple symptoms, such as cognitive, behavioral, and emotional deficits, all of which were often reported by the troops in the Gulf War. A chemical-induced animal model analogous to the Gulf War illness showed an impaired lower nociceptive threshold. VNS restored the impaired nociceptive threshold, relieved anxiety, and improved cognitive deficits in tasks involving object location and pattern separation. VNS significantly reduced neuroinflammation and astrocyte number in the hippocampal CA1, CA3, and dentate gyrus hilus. Meanwhile, VNS improved neurogenesis, with increased doublecortin-expressing cells in the hippocampal dentate gyrus compared with the control group (Nizamutdinov et al., 2018; Venkatasamy et al., 2021; Iannucci et al., 2022).

#### 4.1.6. Epilepsy

Recent human studies have shown inconclusive results on the immunomodulatory effects of VNS in patients with DRE. In a pilot study of five pediatric patients with DRE implanted VNS, the variations of IgA, IgE, IgG, CD19, and pentraxin-3 (PTX-3) displayed a tendency toward a positive correlation between pre-surgery and 6 weeks after VNS. IL-1β and PTX-3 levels tended to decrease more in patients with >80–100% seizure reduction, whereas TNF-α levels decreased in patients with 60–79% seizure reduction and slightly increased in patients with >80–100% seizure reduction at 6 weeks after VNS. However, these results did not reach statistical significance, which may require a larger sample size (Baro et al., 2022). Another postmortem study recruited four patients with VNS and four without VNS as controls. Three of the four VNS-treated patients and all four control patients without VNS died from a sudden unexpected death in epilepsy (SUDEP). There was no laterality difference in the expressions of neuronal nuclear protein, glial fibrillary acidic protein, human leukocyte antigen (HLA)-DR isotype, and Iba-1 in the LC and NTS of VNS-treated patients. Similarly, there was no difference in the rostral pontine group of the raphe nuclei, LC, and NTS between the VNS-treated and non–VNS-treated groups (Ding et al., 2021). Kaur et al. analyzed the gene expression in peripheral blood mononuclear cells in patients with DRE treated with VNS compared with the non–VNS-treated groups. They reported that genes related to stress, inflammatory response, and immunity were downregulated in VNS-treated patients, suggesting an anti-inflammatory effect of VNS for patients with epilepsy (Kaur et al., 2023).

#### 4.1.7. Spinal cord injury

In a rat model of spinal cord injury (SCI), the VNS-treated group exhibited better functional recovery, with reduced glial and fibrotic scar formation and tissue damage. The beneficial effects of VNS were diminished after α7nAChR blockade. VNS inhibited the pro-inflammatory cytokines TNF-α, IL-1β, and IL-6 and increased the expression of IL-10 in the injured spinal cord. In Iba-1-stained microglia, several protein markers can recognize its pro-inflammatory M1 and anti-inflammatory M2 polarization phenotypes, e.g., CD16, CD32, CD40, and CD86 for M1 whereas CD206 and CD163 for M2 (Jurga et al., 2020). VNS promoted the shift of M1-polarized Iba-1^+^/CD86^+^ microglia to M2-polarized Iba-1^+^/CD206^+^ microglia via upregulating α7nAChR to alleviate the neuroinflammation after SCI (Chen et al., 2022).

#### 4.1.8. Chemotherapy-induced polyneuropathy

Rats receiving chemotherapy with paclitaxel exhibited a decrease in heat and mechanical pain thresholds. The pain threshold in the chemotherapy-treated rats significantly increased on day 1 post-VNS but not on day 7 post-VNS. IL-10 level was elevated in the dorsal root ganglia of rats treated with VNS compared with those treated with sham treatment, whereas no apparent changes in nuclear factor kappa B (NF-κB) or TNF-α levels were observed (Zhang et al., 2020).

### 4.2. Non-invasive VNS

#### 4.2.1. Percutaneous cervical or auricular VNS with needle insertion

*Neuroinflammation-related cognitive decline*: VNS can be delivered using a percutaneous echo-guided needle inserted into the carotid sheath, termed percutaneous cervical VNS. Percutaneous cervical VNS decreased the heart rate and increased c-Fos and choline acetyltransferase expression in the brainstem nuclei. Percutaneous cervical VNS significantly reduced plasma levels of TNF-α at 3 h post-lipopolysaccharide (LPS) injection. Furthermore, it decreased Iba-1^+^ microglia numbers in the hippocampus, altered the morphology of microglia, and restored cognitive dysfunction in animals with LPS-induced neuroinflammation (Huffman et al., 2019).

*Parkinson's disease*: In a study by Jiang et al., percutaneous auricular VNS treatment delivered by acupuncture needle significantly improved motor deficits, increased the expressions of TH in the SN and α7nAChR in the ventral midbrain, and reduced the levels of inflammatory cytokines TNF-α and IL-1β. Additionally, percutaneous auricular VNS increased the number of regulatory T cells while decreasing the number of T helper 17 cells in an animal model of 6-OHDA-induced Parkinson's disease (Jiang et al., 2018). *Post-operative cognitive decline*: Post-operative memory impairment after exploratory laparotomy surgery was alleviated in animals treated with percutaneous auricular VNS. The animals treated with VNS showed shorter swimming latency and distance in Morris water maze tests. Treatment with percutaneous auricular VNS decreased the levels of IL-1β and TNF-α and the nuclear protein expression of NF-κB. *Neurodegenerative pathology*: Neurodegenerative pathology included tau phosphorylation and Aβ40 and Aβ42 expressions in the hippocampus in aged rats (Cai et al., 2019). Percutaneous auricular VNS delivered by an acupuncture needle significantly reversed the abovementioned phenomena; however, improvements in α7nAChR (-/-) rats and rats receiving α7nAChR antagonists were diminished. These findings are consistent with those of the previous studies on the vital role of α7nAChR in the VNS-mediated CAP (Wang J. Y. et al., 2021).

#### 4.2.2. Transcutaneous cervical VNS (tcVNS) on the skin adjacent to the vagal nerve

*Migraine*: In a rat model of migraine, Liu et al. demonstrated that non-invasive nVNS suppressed the susceptibility of cortical spreading depression (CSD) in a stimulation intensity-dependent manner. Low-intensity VNS was ineffective, whereas medium-intensity VNS had an efficacy comparable to that of high-intensity VNS. Two 2-min nVNS spaced 5 min apart showed the highest efficacy on the electrical CSD threshold and frequency of KCl-evoked CSD. The optimal nVNS also attenuated CSD-induced upregulation of cortical cyclooxygenase-2, calcitonin gene-related peptide in trigeminal ganglia, and c-Fos expression in the trigeminal nucleus caudalis (Liu et al., 2022). In a double-blinded, sham-controlled study that enrolled 48 patients with migraine, 2 months of adjunctive cervical nVNS significantly reduced the number of severe headaches per month. Pro-inflammatory IL-1β plasma levels (interictal) were higher in the sham-treated patients compared with the nVNS-treated group. However, pro- (IL-6, HMGB1, TNF-a, and leptin) and anti-inflammatory (IL-10, adiponectin, and ghrelin) mediators did not significantly differ between the nVNS and sham groups (Chaudhry et al., 2019).

#### 4.2.3. Transcutaneous auricular VNS (taVNS)

Most of the commercial devices of taVNS are applied at the concha of the external ear, but it is not exclusively stimulating the vagus nerve for complicated nerve distributions. The anterior and superior parts are innervated by auriculotemporal nerve, a sensory branch of the mandibular nerve. The external acoustic meatus is innervated by the facial nerve and auricular branch of the vagus nerve. The inferior and posterior parts of the external ear are innervated by the greater auricular and lesser occipital nerves, which are superficial branches of the cervical plexus with fibers from the C2 and C3 spinal nerves. The precise cutaneous map remains unclear because the cutaneous distribution of a particular nerve varies in some cases, and the boundaries between particular dermatomes often overlap. Moreover, some nerve fibers may cross-communicate with others along their intracranial course (Butt et al., 2020). *Depression*: Wang et al. demonstrated that depression-like behavior and hippocampal neuroinflammation developed in rats with chronic, unpredictable, and mild stress-induced depression. Downregulated expression of α7nAChR, upregulated expression of NF-κB, p65, and IL-1β, and microglial morphology changes in amoebic-like activated states were observed in the depressed rats (Wang J. Y. et al., 2021). Guo et al. provided a novel approach to delivering transcutaneous auricular VNS (taVNS) by a self-adsorptive magnet fixed in the bilateral cavities of the auricular concha. The levels of plasma TNF-α and the expression of TNF-α in the prefrontal cortex, hippocampus, amygdala, and hypothalamus increased in depressed animals. taVNS showed a trend of decreased weight gain, improved sucrose preference, increased mechanical withdrawal threshold, and suppression of elevated TNF-α levels in rats with depression and chronic somatic pain (Guo et al., 2020). These studies indicate that taVNS exerts antidepressive effects via an α7nAChR-dependent mechanism to modulate TNF-α and microglia-mediated neuroinflammation.

## 5. Clinical implications of VNS

**Figure 2:**
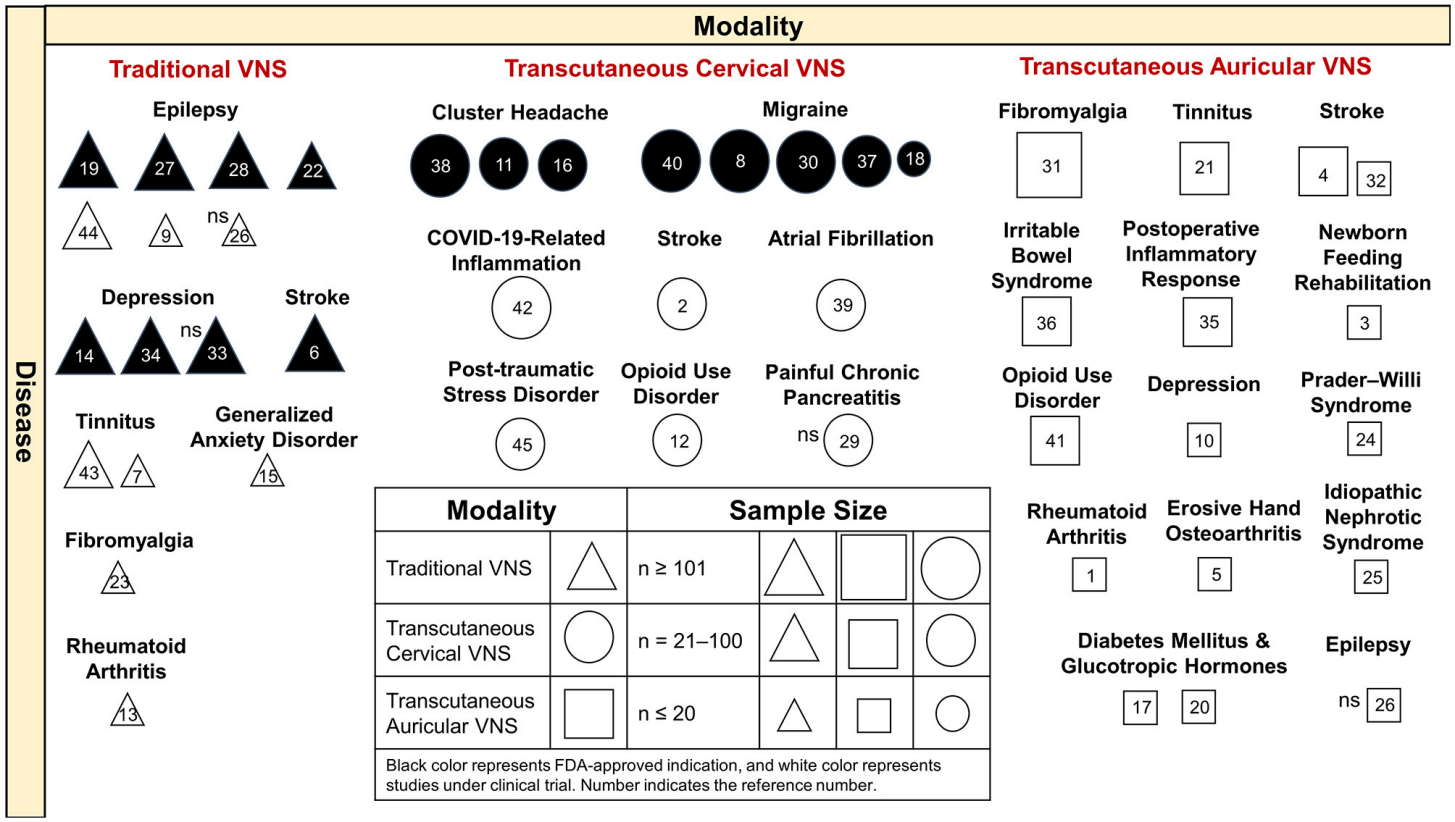
Clinical implications of VNS by different modalities and diseases. Traditional VNS is applied in the treatment of refractory epilepsy and depression and stroke, with FDA-approved indications. VNS is undergoing clinical trials for the treatment of epilepsy, tinnitus, generalized anxiety disorders, fibromyalgia, and rheumatic arthritis. Transcutaneous cervical VNS is FDA approved for cluster headache and migraine and is under clinical trials of COVID-19-related inflammation, stroke, atrial fibrillation, post-traumatic stress disorder, opioid use disorder, and painful chronic pancreatitis. Transcutaneous auricular VNS was applied in a broad spectrum of clinical studies. Most of the VNS studies have achieved the primary outcome, with significant effects, except for references 26, 29, and 33. VNS, vagus nerve stimulation; FDA, Food and Drug Administration; COVID-19, coronavirus disease 2019; ns, non-significant effect on study measure outcome. **Reference number**, 1 (Addorisio et al., 2019), 2 (Arsava et al., 2022), 3 (Badran et al., 2020), 4 (Chang et al., 2021), 5 (Courties et al., 2022), 6 (Dawson et al., 2021), 7 (De Ridder et al., 2015), 8 (Diener et al., 2019), 9 (Fisher et al., 2016), 10 (Garcia et al., 2021), 11 (Gaul et al., 2016), 12 (Gazi et al., 2022), 13 (Genovese et al., 2020), 14 (George et al., 2005), 15 (George et al., 2008), 16 (Goadsby et al., 2018), 17 (Gomolka et al., 2018), 18 (Grazzi et al., 2017), 19 (Kawai et al., 2017), 20 (Kozorosky et al., 2022), 21 (Kreuzer et al., 2014), 22 (Labar et al., 1999), 23 (Lange et al., 2011), 24 (Manning et al., 2019), 25 (Merchant et al., 2022), 26 (Mertens et al., 2022), 27 (Morris et al., 2013), 28 (Morris and Mueller, 1999), 29 (Muthulingam et al., 2021), 30 (Najib et al., 2022), 31 (Paccione et al., 2022), 32 (Redgrave et al., 2018), 33 (Rush et al., 2005a), 34 (Rush et al., 2005b), 35 (Salama et al., 2020), 36 (Shi et al., 2021), 37 (Silberstein et al., 2016a), 38 (Silberstein et al., 2016b), 39 (Stavrakis et al., 2020), 40 (Tassorelli et al., 2018), 41 (Tirado et al., 2022), 42 (Tornero et al., 2022), 43 (Tyler et al., 2017), 44 (Verrier et al., 2016), and 45 (Wittbrodt et al., 2021).

We introduced the FDA-approved indications and the extended clinical trials of VNS based on the discussed diseases. We summarized the modalities, diseases, study sample sizes, and indications with FDA approval or under clinical trial investigation in an integrated figure to provide an overview of the clinical implications of VNS (Figure 2).

### 5.1. FDA-approved indications of VNS

The FDA has approved the use of traditional VNS as adjunctive therapy for patients with DRE and treatment-resistant depression and paired use with post-stroke rehabilitation. nVNS has been approved for the acute treatment of episodic CH and episodic migraines and the prevention of chronic CH and episodic or chronic migraines (Table 3).

**Table 3:** FDA approved indications of VNS.

**VNS Modality**	**Disease**	**Year**	**Population**	**Reference**
Traditional VNS	Drug-resistant epilepsy	1997	Adults and adolescents aged 12–17 years	Morris and Mueller (1999)
	Drug-resistant epilepsy	2017	Children aged ≥ 4 years	Labar et al. (1999) Morris et al. (2013) Kawai et al. (2017)
	Treatment-resistant depression	2005	Adults	Rush et al. (2005a) George et al. (2005) Rush et al. (2005b)
	Post-ischemic stroke rehabilitation	2021	Adults	Dawson et al. (2021)
Transcutaneous cervical VNS	Acute treatment of episodic cluster headache	2017	Adults	Silberstein et al. (2016b) Goadsby et al. (2018)
	Prevention of chronic cluster headache	2018	Adults	Gaul et al. (2016)
	Acute treatment of episodic migraine	2018	Adults	Tassorelli et al. (2018)
	Acute treatment of episodic migraine	2021	Adolescent 12-17 years old	Grazzi et al. (2017)
	Prevention of episodic or chronic migraine	2021	Adults and adolescents aged 12–17 years	Silberstein et al. (2016a) Diener et al. (2019) Najib et al. (2022)

#### 5.1.1. Drug-resistant epilepsy

VNS was first approved as an adjunctive therapy in adults and adolescents aged 12–17 years with DRE in 1997, based on the results of the clinical trials E-01, E-02, E-03, E-04, and E-05 by Morris and Mueller (1999). A total of 454 patients were implanted with VNS, among whom 440 had assessable data. A ≥50% seizure reduction post-implantation was observed in 36.8% of the patients at 1 year, 43.2% of the patients at 2 years, and 42.7% of the patients at 3 years. Median seizure reductions compared with baseline were 35% at 1 year, 44.3% at 2 years, and 44.1% at 3 years. Continuation with VNS rates were 96.7% at 1 year, 84.7% at 2 years, and 72.1% at 3 years. The device was safe and well-tolerated. In 2017, the FDA approved VNS for patients aged 4–11 years with DRE. In the subgroup analysis of the E-04 trial by Labar et al., 24 patients aged > 2 years with drug-resistant generalized epilepsy were prospectively enrolled. The median seizure rate reduction was 46%. Sixteen out of the 24 patients had a > 30% reduction in seizure rate, and 11 had a > 50% reduction. In this cohort, patients with higher baseline seizure rates and later-onset age of epilepsy had the best response to VNS. Furthermore, VNS was an effective treatment for DRE even in patients as young as 4 years old (Labar et al., 1999). According to the evidence-based guideline published by Morris et al. (2013). VNS was possibly effective in achieving >50% seizure frequency reduction, based on the data from 14 Class III studies. In a pooled analysis of data from 481 children, the responder rate was 55%. A Japanese post-approval study published by Kawai et al. prospectively enrolled 78 patients aged <12 years treated with VNS. The median decreases in all the seizures after 3, 6, 12, 24, and 36 months following VNS therapy were 9.0%, 40.2%, 50.0%, 50.0%, and 60.0%, respectively (Kawai et al., 2017).

#### 5.1.2. Treatment-resistant depression

In 2005, the FDA approved the use of VNS for treatment-resistant depression, based on the randomized, double-blind D-02 trial. In the acute phase of the trial, there were no significant differences in the 24-item Hamilton Rating Scale for Depression (HRSD-24) and Inventory of Depressive Symptomatology Self-Report (IDS-SR30) after 10 weeks of VNS stimulation compared with the sham group (Rush et al., 2005a). Twelve months after VNS implantation, there was a significant reduction in HRSD-24. The response rate measured by the HRSD-24 was 27.2%, and the remission rate was 15.8%. Similar results were obtained with the Montgomery Asberg Depression Rating Scale (28.2% response rate) and Clinical Global Impression-Improvement scale (34.0% response rate) after 12 months of VNS. Voice alteration, dyspnea, and neck pain were the most frequently reported adverse events (Rush et al., 2005b). Furthermore, compared with the treatment-as-usual (TAU) group, the VNS + TAU group was associated with greater improvement per month in the IDS-SR30. The response rates of the HRSD-24 were 27% for the VNS + TAU group and 13% for the TAU group (George et al., 2005).

#### 5.1.3. Acute treatment of episodic cluster headache

The FDA has approved nVNS for the acute treatment of episodic CHs, based on the ACT1 and ACT2 trials. The ACT1 trial by Silberstein et al. included 133 patients with CH randomized into an nVNS-treated group (60 patients: episodic CH, *n* = 38; chronic CH, *n* = 22) and a sham-treated group (73 patients: episodic CH, *n* = 47; cluster CH, *n* = 26). Response rates were significantly higher with nVNS than with a sham treatment in the episodic CH cohort (nVNS, 34.2%; sham, 10.6%) but not in the chronic CH cohort (nVNS, 13.6%; sham, 23.1%). Sustained response rates were significantly higher with nVNS for the episodic CH cohort and total population (Silberstein et al., 2016b). The ACT2 trial by Goadsby et al. included 48 nVNS-treated (14 episodic CH and 34 chronic CH) and 44 sham-treated patients (13 episodic CH, 31 chronic CH). Regarding the proportion of all treated attacks that achieved a pain-free status within 15 min after treatment initiation, nVNS (14%) and sham (12%) treatments were not significantly different for the total cohort. However, nVNS (48%) was superior to sham treatment (6%, *p* < 0.01) in the episodic CH subgroup. No significant differences between the nVNS (5%) and sham (13%) treatments were observed in the chronic CH subgroup (Goadsby et al., 2018).

#### 5.1.4. Acute treatment of episodic migraine

In 2018, nVNS was approved for the acute treatment of episodic migraine, based on the double-blind, randomized PRESTO study. A total of 248 participants with episodic migraine with/without aura were randomized to receive nVNS or sham stimulation within 20 min of pain onset. Results showed that nVNS (*n* = 120) was superior to sham treatment (*n* = 123) for pain freedom at 30 min (12.7% vs. 4.2%, *p* = 0.012) and 60 min (21.0% vs. 10.0%, *p* = 0.023) but not at 120 min after the first treated attack. A *post hoc* repeated-measures test showed the therapeutic benefit of nVNS at 30, 60, and 120 min (odds ratio, 2.3; 95% confidence interval, 1.2–4.4; *p* = 0.012). nVNS demonstrated benefits across other endpoints, including pain relief at 120 min, and was safe and well-tolerated (Tassorelli et al., 2018). In 2021, the FDA approved nVNS for the acute treatment of episodic migraine in adolescents, based on the results of the study of Grazzi et al., which enrolled 47 adolescent patients with migraine without aura. Of the treated migraine attacks, 46.8% were considered successfully treated and did not require any rescue medication. No device-related adverse events were recorded (Grazzi et al., 2017).

#### 5.1.5. Prevention of chronic cluster headache

In 2018, the FDA approved nVNS for the prevention of chronic CHs, based on the results of the PREVA trial. The PREVA trial was an open-label randomized study conducted by Gaul et al. Patients with chronic CHs were randomized into the adjunctive prophylactic nVNS (*n* = 48) or standard of care (SoC) alone (control, *n* = 49) group. During the randomized phase, the SoC + nVNS group had a significantly greater reduction in the number of attacks per week compared with the controls, with a mean therapeutic gain of 3.9 fewer attacks per week. A greater percentage of ≥50% response rates was also observed in the SoC + nVNS (40%) group compared with the control group (8.3%) (Gaul et al., 2016).

#### 5.1.6. Prevention of episodic or chronic migraine

In 2021, the FDA approved nVNS for adults and adolescents aged 12–17 years for the prevention of episodic and chronic migraines, based on the result of the EVENT, PREMIUM I, and PREMIUM II trials. In the EVENT trial, 59 adults with chronic migraines were enrolled. The mean changes in the number of headache days were −1.4 with nVNS and −0.2 with sham treatment, without significant statistical differences. Among the 27 participants who completed the open-label phase, 15 were initially assigned to nVNS, and their mean change from baseline in headache days after 8 months of treatment was −7.9 (95% confidence interval, −11.9 to −3.8; *p* < 0.01) (Silberstein et al., 2016a). In the PREMIUM trial, 477 adults with episodic migraines were enrolled. Mean reductions in migraine days per month were not significantly different from those in the sham group. *Post hoc* analysis of patients with ≥ 67% adherence per month demonstrated a significant difference between nVNS and sham for the reduction in migraine days (2.27 vs. 1.53, *p* = 0.043). Patients with migraine with aura responded better than those with migraine without aura (Diener et al., 2019). In the PREMIUM II trial, 336 adults with episodic or chronic migraines were enrolled. There was a significant mean reduction in monthly migraine days in the nVNS group compared with the sham group (3.12, 2.29, difference, −0.83, *p* = 0.2329). The responder rate was greater in the nVNS group (44.87%) than in the sham group (26.81%, *p* = 0.0481) (Najib et al., 2022).

#### 5.1.7. Paired with post-ischemic stroke rehabilitation

The VNS-REHAB trial was a pivotal, randomized, triple-blinded, and sham-controlled study. This trial enrolled 108 adults at least 9 months after an ischemic stroke with moderate-to-severe arm weakness. The patients were assigned to the rehabilitation paired with active VNS (VNS group, *n* = 53) or rehabilitation paired with sham stimulation (sham group, *n* = 55) groups. Among the 106 patients who completed the study, there was a significant improvement in the mean Fugl–Meyer Assessment-Upper Extremity (FMA-UE) score in the VNS group compared with the control group. A clinically meaningful response in the FMA-UE score was achieved in 23 (47%) out of 53 patients in the VNS group vs. 13 (24%) out of 55 patients in the control group 90 days after in-clinic therapy, which was significantly greater in the VNS group compared with the control group (between-group difference, 24%; *p* = 0.0098) (Dawson et al., 2021).

### 5.2. Clinical trials of VNS with published results

Currently, there are several ongoing studies on the clinical utility of VNS. We reviewed recently completed clinical trials with available published data to provide a scope for current practice, off-label use, and unmet needs (Table 4).

**Table 4:** Clinical trials on VNS with published results.

**VNS Modality**	**Disease**	**Trial identifier**	**Reference**
Traditional VNS	Epilepsy	NCT01846741	Fisher et al. (2016)
		NCT01325623	Verrier et al. (2016)
		NCT05031208	Mertens et al. (2022)
	Tinnitus	NCT01253616	De Ridder et al. (2015)
		NCT01962558	Tyler et al. (2017)
	Fibromyalgia	NCT00294281	Lange et al. (2011)
	Generalized anxiety disorder	NCT03440255	George et al. (2008)
	Rheumatoid arthritis	NCT03437473	Genovese et al. (2020)
Transcutaneous auricular VNS	Epilepsy	NCT05031208	Mertens et al. (2022)
	Tinnitus	NCT01176734	Kreuzer et al. (2014)
	Post-stroke rehabilitation	NCT03170791	Redgrave et al. (2018)
		NCT03592745	Chang et al. (2021)
	Feeding rehabilitation for newborns	NCT04643808	Badran et al. (2020)
	Type 2 diabetes mellitus	NCT02098447	Gomolka et al. (2018)
	Glucotropic and orexigenic hormones	NCT04926415	Kozorosky et al. (2022)
	Fibromyalgia	NCT03180554	Paccione et al. (2022)
	Prader–Willi syndrome	NCT03689621	Manning et al. (2019)
	Major depressive disorder	NCT04467164	Garcia et al. (2021)
	Opioid use disorder	NCT04075214	Tirado et al. (2022)
	Rheumatoid arthritis	NCT01569789	Addorisio et al. (2019)
	Post-operative inflammatory response	NCT03204968	Salama et al. (2020)
	Erosive hand osteoarthritis	NCT03919279	Courties et al. (2022)
	Idiopathic nephrotic syndrome	NCT04169776	Merchant et al. (2022)
	Constipation-predominant irritable bowel syndrome	NCT05392439	Shi et al. (2021)
	Healthy participants, heart rate	NCT02835885	Badran et al. (2018)
	Healthy participants, motor performance	NCT04768738	Hatik et al. (2022)
	Healthy participants, sleep	NCT04070547	Jackowska et al. (2022)
	Healthy participants, gastric motility	NCT02359188	Steidel et al. (2021)
	Healthy participants, intestinal barrier dysfunction	NCT04061564	Mogilevski et al. (2022)
Transcutaneous cervical VNS	Acute stroke	NCT03733431	Arsava et al. (2022)
	Atrial fibrillation	NCT02548754	Stavrakis et al. (2020)
	Post-traumatic stress disorder	NCT02992899	Wittbrodt et al. (2021)
	Opioid use disorder	NCT04556552	Gazi et al. (2022)
	COVID-19-related inflammation	NCT04368156	Tornero et al. (2022)
	Painful chronic pancreatitis	NCT03357029	Muthulingam et al. (2021)
Transvenous VNS	Healthy participants, inflammatory response	NCT01944228	Kox et al. (2015)

#### 5.2.1. Physiological functions in healthy participants

Some trials were conducted on healthy participants to understand the mechanism of action of VNS and its effect on the immune response. In healthy participants, transvenous VNS was insufficient to modulate the innate immune response during endotoxemia (Kox et al., 2015). Short-term non-invasive auricular VNS did not significantly affect the motor performance of young healthy individuals in the cycle ergometer test (Hatik et al., 2022). Short trains of taVNS decreased the heart rate in a stimulation parameter-dependent manner (Badran et al., 2018). taVNS showed a trend in improving the global sleep score and significantly improving gastric motility (Steidel et al., 2021; Jackowska et al., 2022). Transcutaneous VNS was applied to test its protective effect on stress-induced intestinal barrier dysfunction. A reduction in paracellular permeability of the small intestine was observed in the VNS-treated group measured by intestinal fatty acid-binding protein release, although it did not entirely mitigate intestinal barrier dysfunction (Mogilevski et al., 2022).

#### 5.2.2. Epilepsy

In patients with epilepsy, traditional VNS with an open-loop system has been shown with safety, tolerability, and a significant seizure reduction rate (Batson et al., 2022). The closed-loop device of VNS equipped with seizure detection and automatic stimulation mode upon sensing tachycardia also showed beneficial effects in patients with epilepsy (Fisher et al., 2016). Furthermore, VNS may reduce the interictal cardiac electrical instability and T-wave alternans in patients with DRE and may further provide better prevention of SUDEP (Verrier et al., 2016). Mertens et al. used traditional or taVNS in patients with DRE to investigate the effects of VNS on verbal memory. There was improved verbal memory performance after 6 weeks of traditional VNS treatment. Acute VNS and taVNS did not improve verbal memory performance (Mertens et al., 2022). Currently, the efficacy of tcVNS and taVNS on DRE remained to be elucidated. Several compelling studies have suggested that stimulating large-diameter afferent myelinated fibers by traditional VNS can alter the activation and connectivity of the neural network through the vagal–NTS–parabrachial nucleus–hypothalamus–thalamus–limbic system to the cortex and induce catecholamine releases, whereby VNS effectively reduces seizures in patients with DRE (Carron et al., 2023).

#### 5.2.3. Tinnitus

Regarding tinnitus, variable results of efficacy were reported due to the small number of patients and heterogeneity of the study design. One investigation of taVNS for the control of tinnitus showed no improvement after 6 months of stimulation (Kreuzer et al., 2014). In another case report by De Ridder et al. (2015) a patient had improvement of tinnitus after VNS (paired with tones) and had a recurrence of tinnitus after sham stimulation. In a small, double-blind, control pilot trial, Tyler et al. found improvement in tinnitus in patients receiving VNS paired with tone but not in the VNS-only-treated group, particularly with tonal and non–blast-induced tinnitus (De Ridder et al., 2015; Tyler et al., 2017).

#### 5.2.4. VNS paired rehabilitation

Regarding stroke, VNS is thought to enhance the task-related circuit plasticity, thus facilitating post-stroke rehabilitation to improve motor, speech, and swallowing functions (Morrison et al., 2021). Redgrave et al. demonstrated the feasibility and safety of taVNS with upper limb repetitive movement for post-stroke rehabilitation (Redgrave et al., 2018). taVNS with upper limb robotic training reduced the spasticity and variability of the biceps brachii surface electromyography peak amplitude in extension movements (Chang et al., 2021). Furthermore, one pilot study by Arsava et al. (2022) showed feasibility, safety, and a reduction of stroke size via tcVNS in the setting of acute ischemic and hemorrhagic stroke. Badran et al. (2020) found that 57% of enrolled premature infants or infants with hypoxic–ischemic encephalopathy and oromotor dysfunction who received taVNS-paired feeding rehabilitation achieved adequate feeding volumes without the need for G-tube insertion at discharge, with a significant increase in feeding volume compared with pre-stimulation.

#### 5.2.5. Metabolic and cardiac diseases

taVNS was found to lower postprandial plasma ghrelin levels in healthy participants receiving a high-calorie beverage, which posed its probable therapeutic application for reducing oral intake in patients with diabetes mellitus (Kozorosky et al., 2022). Gomolka et al. (2018) demonstrated that the Higuchi fractal dimension method on HRV could readily differentiate patients with diabetes mellitus from normal controls and could provide stimulation feedback for taVNS. VNS exerts a variety of influences on heart rate and HRV among different study populations with variable stimulation modalities, sites, and parameters (Dolphin et al., 2022). It may have therapeutic implications for patients with cardiovascular diseases, such as heart failure and arrhythmia (Kharbanda et al., 2022; Konstam et al., 2022; Verrier et al., 2022). tcVNS reduced the burden of atrial fibrillation after 6 months of daily treatment (Stavrakis et al., 2020).

#### 5.2.6. Chronic stress, mood, pain, and cognition

Chronic stress may aggravate pre-existing psychiatric diseases, and VNS provides an opportunity to treat such diseases by the augmentation of the HPA axis. In patients with generalized anxiety disorder, a pilot study by George et al. showed the tolerability of VNS treatment with the evidence of acute and long-term improvement in some patients (George et al., 2008). In patients with fibromyalgia, Lange et al. demonstrated the feasibility and tolerability of VNS (Lange et al., 2011). taVNS improved overall fibromyalgia severity by clinical evaluation. However, there were no significant differences in the visual analog scale rated by patients or HRV compared with the sham group (Paccione et al., 2022). Wittbrodt et al. (2021) recruited healthy participants with traumatic experiences and found that tcVNS increased anterior cingulate and hippocampal activity when exposed to trauma scripts. Transcutaneous VNS was effective in reducing temper outbursts in Prader–Willi syndrome (Manning et al., 2019). In contrast to traditional VNS with FDA-approved indication for the treatment of patients with refractory depression, the effects of taVNS have been explored in patients with depression (Austelle et al., 2022). Recently, a novel device with respiratory-gated auricular vagal afferent nerve stimulation (RAVANS) effectively modulated the brain circuitries that regulate the response to negative stress. RAVANS was associated with a significant acute reduction in symptoms of depression and anxiety in women with recurrent major depressive disorder (Garcia et al., 2021). Regarding opioid withdrawal syndrome, studies have also demonstrated that tcVNS may decrease subjective opioid withdrawal, reduce distress, and lower the heart rate during acute opioid withdrawal (Gazi et al., 2022; Tirado et al., 2022). Based on the rationale that VNS can activate the LC and catecholamines in the hippocampus and cortex, VNS undergoes some clinical trials to improve cognitive functions in patients with Alzheimer's disease, but these trials did not achieve a conclusive significance (Vargas-Caballero et al., 2022).

#### 5.2.7. Systemic inflammatory and autoimmune-related diseases

VNS is possibly therapeutic in the field of systemic inflammatory response, including sepsis and autoimmune diseases. In patients with multidrug-refractory rheumatoid arthritis, traditional VNS showed effectiveness in reducing signs and symptoms (Genovese et al., 2020). taVNS also showed efficacy in the inhibition of peripheral blood production of TNF, IL-1β, and IL-6 in healthy participants and attenuated systemic inflammatory responses in patients with rheumatoid arthritis (Addorisio et al., 2019). A recent randomized control trial enrolled patients with coronavirus disease 2019 (COVID) and found that tcVNS significantly reduced the levels of inflammatory markers, specifically C-reactive protein (CRP) and procalcitonin (Czura et al., 2022; Tornero et al., 2022). Post-operative taVNS after lung lobectomy attenuated the acute postsurgical inflammatory response by regulating IL-6 and IL-10, resulting in reduced incidence of Post-operative pneumonia and hospitalization time (Salama et al., 2020). In a pilot study of erosive hand osteoarthritis, taVNS significantly reduced the hand pain in the visual analog scale score and functional index for hand arthropathy score (Courties et al., 2022). In a small trial that included patients with idiopathic nephrotic syndrome, taVNS was associated with clinical remission in frequently relapsing nephrotic syndrome and reduced proteinuria in non-congenital steroid-resistant nephrotic syndrome (Merchant et al., 2022). In constipation-predominant irritable bowel syndrome, 4 weeks of taVNS improved both constipation and abdominal pain (Shi et al., 2021). However, the pain-relieving effect was not observed in patients with painful chronic pancreatitis receiving tcVNS (Muthulingam et al., 2021).

## 6. Conclusion and perspectives

The neuroimmunomodulation effect of VNS is mediated through the CAP, which is an α7nAChR-dependent pathway that reduces pro-inflammatory cytokines. Studies on the mechanisms of VNS included *in vivo* or *in vitro* experiments, and the therapeutic effects were verified in animal studies with a broad spectrum of diseases, including systemic diseases with inflammatory pathophysiology and neuropsychiatric diseases. However, whether the immunomodulating effects shown in animal studies can be reproduced in humans and particularly patients requires further evidence. nVNS is currently being explored in device developments and clinical applications to examine whether the immunomodulation in traditional VNS can be repeated using nVNS as the stimulation modalities and protocols are different from those in traditional VNS. Large prospective randomized controlled trials are required to elucidate the optimal stimulation parameters. Moreover, the acute and long-lasting effects of VNS should be carefully monitored as chronic stimulation would change neural plasticity and excitability. Furthermore, the vagus nerve connects the brain and internal organs. This opens up a unique opportunity to investigate the bidirectional interactions of cognition, mood, and systemic diseases with VNS modulation, e.g., the emerging topics in the gut–vagus–brain axis in inflammatory and neuropsychiatric diseases. VNS activates both vagal afferent and efferent fibers. However, most of the knowledge on the neuroimmunomodulatory effects of VNS involves the vagal efferent pathway and its related peripheral mechanisms. Little is known about how the brain is activated by the vagal afferent pathway and its relationship to the modulation of neuroendocrine and immune systems. Therefore, further studies are warranted to elucidate the neural substrates and neurophysiological mechanisms of VNS via the vagal afferent pathway to the brain and their contributions to regulating the inflammatory process that underlies the therapeutic effects of VNS.

## Author contributions

Y-TF, Y-TL, W-LT, G-LH, PT, Y-JC, and Y-JW wrote the manuscript. Y-JW organized the manuscript. PT and Y-JW edited the manuscript. Y-TL and W-LT drew the figures. Y-TF generated the tables. All authors contributed to the article and approved the submitted version.
